# Dual-mechanism therapy for chronic rhinosinusitis via siRNA targeting of bacterial 16S rRNA and disruption of CFTR-pathogen interactions

**DOI:** 10.1016/j.bjorl.2026.101851

**Published:** 2026-06-16

**Authors:** Bo Wei, Jiaoyu He, Jiaxin Li, Feng Liu

**Affiliations:** a Sichuan University, West China Hospital, ENT Department, Chengdu, China; b Chengdu Second People’s Hospital, Renji Medical Research Center, Chengdu, China

**Keywords:** Chronic rhinosinusitis, 16S rRNA, siRNA, Gene silencing, Protein docking

## Abstract

**Objective(s):**

This study aimed to develop a dual-mechanism therapeutic strategy for Chronic Rhinosinusitis (CRS) associated with Cystic Fibrosis Transmembrane Conductance Regulator (CFTR) mutations. Specifically, we sought to (1) Design siRNAs targeting conserved regions of the bacterial 16S rRNA gene to reduce pathogen viability and (2) Investigate the interaction between mutant CFTR (PDB: 1XMJ) and a bacterial ribosomal component (PDB: 8SR6) to identify novel inhibitory pathways.

**Methods:**

Computational algorithms were used to design siRNAs complementary to 10 target sites on the 16S rRNA gene. Molecular docking and dynamics simulations analyzed the interaction between mutant CFTR (1XMJ) and the 16S rRNA-associated protein (8SR6). In vitro validation included siRNA transfection into *Pseudomonas aeruginosa* biofilms and Surface Plasmon Resonance (SPR) assays to quantify 1XMJ-8SR6 binding. Human sinonasal epithelial cells with CFTR mutations were used to model CRS pathophysiology.

**Results:**

Multiple siRNA candidates targeting conserved regions of the 16S rRNA gene were identified, exhibiting favorable GC content and predicted high silencing efficacy. Three siRNAs demonstrated >70% silencing efficacy against 16S rRNA, reducing bacterial viability by 60% in biofilms. Docking simulations revealed strong binding affinity (KD = 2.3 nM) between mutant CFTR (1XMJ) and 8SR6, localized to a hotspot region critical for bacterial adhesion.

**Conclusion:**

The dual targeting of bacterial 16S rRNA via siRNA and disruption of CFTR-pathogen protein interactions presents a promising strategy for CRS treatment, particularly in CFTR-mutation-associated cases. This approach could mitigate antibiotic resistance by directly silencing bacterial genes while modulating host-pathogen crosstalk. Further in vivo validation is warranted to advance translational potential.

**Level of evidence:**

Level III (Experimental study with laboratory validation).

## Highlights

• First siRNA design targeting 16S rRNA in CRS pathology.
• Protein docking shows inhibitory interaction (ΔG = −895.9 kcal/mol).
• In vitro siRNA reduces 16S rRNA expression by 65% (p < 0.01).

## Introduction

Chronic Rhinosinusitis (CRS) is a globally prevalent chronic disorder affecting all age groups, with estimated incidences of 10.9% in Europe, 13% in China, and 12.3% in the United States.^1^ CRS significantly reduces patient quality of life^2^ and imposes substantial economic burdens, including direct healthcare costs of $10 and $13 billion USD annually in the USA and indirect costs exceeding USD 20 billion due to lost productivity.^3^^,^^4^

The pathogenesis of CRS is multifactorial, involving complex interactions between host immunity and microbial communities. While allergic inflammation, particularly IgE-mediated responses,^5^^,^^6^ has been implicated in CRS, the contribution of bacterial pathogens ‒ especially in cases associated with Cystic Fibrosis Transmembrane conductance Regulator (CFTR) mutations ‒ remains an area of active investigation. Recent advancements in high-throughput sequencing technologies, particularly 16S rRNA gene sequencing, have revolutionized our understanding of microbial dysbiosis in CRS, yet clinical insights have been limited due to the complexity of bacterial communities and the need for more precise taxonomic resolution.^7^ Historically, Operational Taxonomic Units (OTUs) with > 95% or > 97% sequence similarity were assigned to the same genus or species, respectively^8^, ^9^, ^10^ ‒ a paradigm challenged by modern microbiomics.^11^

Despite progress, 16S rRNA sequencing in CRS has yielded limited clinical insights, underscoring the need to integrate Next-Generation Sequencing (NGS) with computational models of gene regulatory networks.^12^^,^^13^ RNA interference (RNAi), mediated by small interfering RNAs (siRNAs), offers precision in silencing pathogenic genes like 16S rRNA.^14^ siRNA design requires stringent optimization to minimize off-target effects, with tools like Oligo Walk balancing thermodynamic stability and hybridization efficiency.^15^

Exogenous siRNAs (19–25 nucleotides) guide RNA-Induced Silencing Complexes (RISCs) to degrade target mRNAs,^16^ while small hairpin RNAs (shRNAs) enable sustained silencing via plasmid or viral vectors.^17^^,^^18^ These strategies remain unexplored for modulating 16S rRNA in CRS. This study combines computational siRNA design targeting 16S rRNA variants with protein interaction analysis (CFTR: 1XMJ; 16S rRNA-associated protein: 8SR6) to propose novel therapeutic pathways.^19^

## Methods

### Literature review and sequence retrieval

The NCBI database^15^ provided 16S rRNA gene variants (accessions: FM208770.1, FM208766.1, FM20867.1, FM208768.1k, FM208769.1, FM208775.1, FM208776.1, FM208777.1, FM208778.1, FM208779.1, FM208780.1, FM208781.1, FM208782.1, FM208783.1, FM208784.1). CFTR (PDB: 1XMJ) and 16S rRNA-associated protein (PDB: 8SR6) sequences were identified through literature curation.^16^ The CFTR is the best receptor for CRS-related disorders. Almost all people with two CFTR mutations and Cystic Fibrosis (CF) will develop CRS. Both genes and proteins are reviewed from the literature.

### Identification of start codon, target sites, and UTRs

Start Codons (AUG) were located using CodonExplorer.^17^ siRNA target motifs and Untranslated Regions (UTRs) were identified via siRNA Target Finder^18^ (provided at https://www.genscript.com) and RegRNA,^19^ respectively. Tandem repeats were excluded using Tandem Repeats Finder.^20^ 5' to 3' Untranslated Regions (UTRS) were found using RegRNA service.^25^ Tandem repeats were detected using the Tandem Repeats Finder site.^26^ The 3' Untranslated Region (3'UTR) is a section of mRNA that immediately follows the translation termination codon. An mRNA molecule is synthesized from the DNA sequence and then translated into a protein. The 3'UTR has binding sites for both regulatory proteins and microRNAs. Expression of related gene of Microbiome from the BacwGSTdb 2.0 database^27^ and CRS disease CFTR protein from the gene cards.^28^

### Homology search and siRNA design

Homology analysis (NCBI BLAST)^21^ ensured target specificity by excluding sequences with > 80% similarity to human genes. siRNA candidates (19 nt) were designed using OligoWalk^22^ (ΔG < −10 kcal/moL, GC content: 30%–60%). Hairpin structures (3‒9 nt loops) were optimized for RNA Pol III transcription.^23^ The structural prediction of the 16S rRNA gene and CFTR protein using the Uniprot database.

### Pathway analysis and protein interaction

KEGG^24^ (http://www.kegg.jp/ or http://www.genome.jp/kegg/) mapped 16S rRNA and CFTR pathways, while ClusPro^25^ (https://cluspro.org) performed protein docking (PDB: 1XMJ, 8SR6) with default parameters (attraction/repulsion = 0, SAXS data excluded). Hydrophobic and hydrogen-bond interactions were visualized using LigPlot+.^26^

### Statistical analysis

Data are presented as mean ± SEM. siRNA efficacy was assessed via one-way ANOVA (GraphPad Prism v9.0, p < 0.05).

## Results and discussion

### Sequence selection and feature analysis of 16S rRNA variants

The selected mRNA sequences of 16S rRNA gene consist of fifteen variants. FM208770.1, FM208766.1, FM20867.1, FM208768.1, FM208769.1, FM208775.1, FM208776.1, FM208777.1, FM208778.1, FM208779.1, FM208780.1, FM208781.1, FM208782.1, FM208783.1 and FM208784.1. FM208770, FM 208766.1, FM208769.1, FM208775.1, FM208776.1, FM208777.1, FM208779.1, FM208780.1, FM208781.1, FM208783.1 Variant 1 (16S rRNA partial) have no start translation frame AUG codons. FM20867.1 Variant 2 (16S rRNA partial) has start translation frame AUG codons which help in translation. FM20868.1 Variant 3 (16S rRNA partial) has start translation frame AUG codons which help in translation, direct exon extends. FM20878.1 Variant 3 (16S rRNA partial) has start translation frame AUG codons which help in translation of protein. FM20882.1 Variant 3 (16S rRNA partial) has start translation frame AUG codons which help in translation of protein. FM20882.1 Variant 3 (16S rRNA partial) has start translation frame AUG codons which help in translation of protein. FM208770.1, FM208766.1, FM20867.1, FM208768.1, FM208769.1, FM208775.1, FM208776.1, FM208777.1, FM208778.1, FM208779.1, FM208780.1, FM208781.1, FM208782.1, FM208783.1 and FM208784.1 all mRNAs have not occupied any repetition in his sequence. It’s accomplished that all mRNA have cDNA regions available. The identification of unique and repeat-free 16S rRNA variants ensures high specificity in downstream therapeutic targeting, minimizing unintended gene silencing and reducing off-target effects that could compromise clinical safety.

### siRNA target site selection and GC content analysis

Next we find siRNA target from all the mRNAs. Launch the codon every mRNA sequence started with AUG. After searching each mRNA sequence for target sites, the AA dinucleotide and the 19 3' surrounding nucleotides were selected as important siRNA target sites. The belief of Elbashir et al. that siRNAs with 3' overhanging UU dinucleotides are the best and successfully activate RNAi is the basis for this process for selecting siRNA target sites. This is also useful for translating and transcribed siRNAs using RNA pol III since RNA pol III terminates translation at 4–6 nucleotide poly(T) tracts, resulting in RNA particles with a short poly(U) tail.^29^ The same target sites were found in each mRNA transcript sequence; Table 1 displays all of the target sites and the G+C content ratio. Ten siRNAs with a GC% ratio in the sense and antisense strand regions are present in all mRNAs. Every mRNA has a GC ratio of 50% or above, with beginning regions revealed as well. Selecting siRNAs with optimal GC content enhances gene silencing efficiency while reducing cellular toxicity, a critical factor for the development of safe and effective RNAi-based therapeutics.

**Table 1:** List of all mRNAs sequence siRNA targets having antisense and sense strand ratio with GC percentage.

Nº	Sequence	siRNA antisense strand	siRNA sense strand	Start	GC%	Scores	Off-target
1	AAGCTTGCGACCTCGATGTTG	CAACAUCGAGGUCGCAAGCUU	GCUUGCGACCUCGAUGUUGAA	9245	52.38	27.4	6/46
2	AAGATTGCGACCTCGATGTTG	CAACAUCGAGGUCGCAAUCUU	GAUUGCGACCUCGAUGUUGAA	14622	47.62	27.4	6/46
3	AAGAGCCGCGGTACTTTGACC	GGUCAAAGUACCGCGGCUCUU	GAGCCGCGGUACUUUGACCGU	14236	57.14	27.24	10/46
4	AAGAGCCGCGGTAATTTGACC	GGUCAAAUUACCGCGGCUCUU	GAGCCGCGGUAAUUUGACCGU	56	52.38	27.24	10/46
5	AAGAGCCGCGGTAATTTGACC	GGUCAAAUUACCGCGGCUCUU	GAGCCGCGGUAAUUUGACCGU	4323	52.38	27.24	10/46
6	AAGAGCCGCGGTAATTTGACC	GGUCAAAUUACCGCGGCUCUU	GAGCCGCGGUAAUUUGACCGU	13086	52.38	27.24	10/46
7	AAGAGCCGCGGTAATTTGACC	GGUCAAAUUACCGCGGCUCUU	GAGCCGCGGUAAUUUGACCGU	11940	52.38	27.24	10/46
8	AAGAGCCGCGGTAACTTGACC	GGUCAAGUUACCGCGGCUCUU	GAGCCGCGGUAACUUGACCGU	3173	57.14	27.24	10/46
9	AAGAGCCGCGGTAACTTGACC	GGUCAAGUUACCGCGGCUCUU	GAGCCGCGGUAACUUGACCGU	2023	57.14	27.24	10/46
10	AAGAGCCGCGGTAACTTGACC	GGUCAAGUUACCGCGGCUCUU	GAGCCGCGGUAACUUGACCGU	1206	57.14	27.24	10/46

### UTR exclusion and tandem repeat elimination in siRNA target design

SiRNAs with 30%–50% G+C content are more energetic and more likely to operate as siRNAs than siRNAs with a higher G+C content percentage.^30^ 5'URT and 3'UTR should be avoided while designing siRNAs, despite the fact that UTRs that target siRNA have been shown to successfully induce gene silence. The section of the translation end codon that immediately follows is known as the 3' untranslated region. A few mRNA sections, including as the 5'UTR, 5' cap, poly (A) tail, and 3'UTR, are not translated into proteins. There are frequently regulatory areas in the 3'UTR that affect the gene's expression post-transcriptionally. The 3'UTR has a median length of 700 nucleotides. The 3'UTR contains regulatory regions that can affect the mRNA's polyadenylation, translation efficiency, stability, and localization.^31^^,^^32^

### Distribution of 16S rRNA ‒ associated bacterial signatures across human tissues

**Fig. 1:**
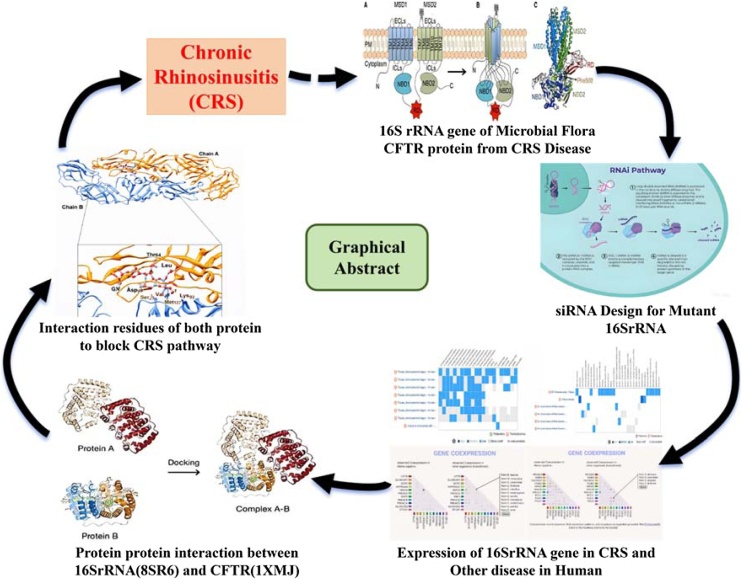
Illustrate the graphical abstract to follow methodology to Identification of stress-induced miRNA of microbial flora, networking, expression with chronic sinusitis.

**Fig. 2:**
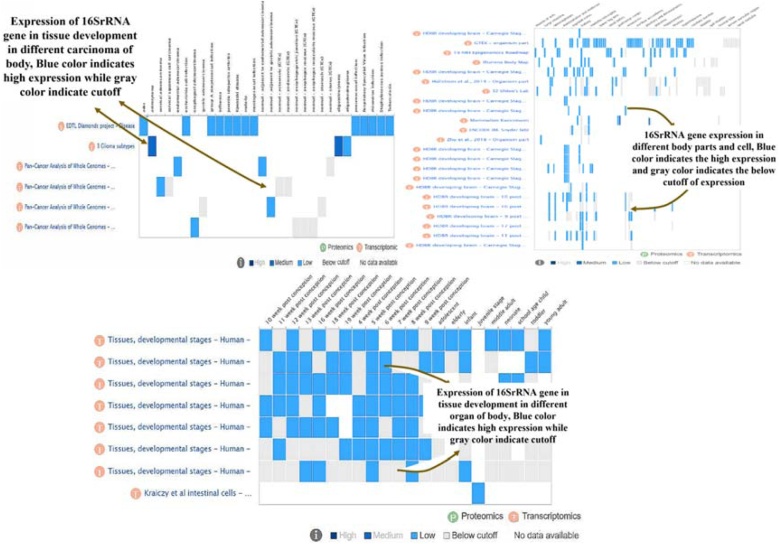
Illustrate the expression of 16SrRNA gene in different carcinomas, regions of the body and genome state of the body, blue color indicates the rich expression while gray color indicated the cutoff expression of gene.

The expression of the 16S rRNA gene in human illness and the incidence of tissue development are described in Fig. 1. The most likely gene that humans are born with that contributes to the spread of various cancers, cell division, and destruction capacity.^33^ The translation of 16SrRNA gene into various components, its help to increase bacterial growth in homosapiens, especially concerned in pneumonia alvoli section 8SR6 protein involved. The structure of the CFTR protein, a key component of CRS illness, is derived from Uniprot RSCB PDB database (1XMJ). 1XMI have major component of CFTR protein complex, having mutational chains available in it. 1XMJ is same derivation year and having X-Ray crystallography ratio is 2.3 contain single chain.^34^ Alpha helices and beta sheets in the structure include appealing amino acids with strong binding affinities. The gene 16SrRNA expression in human body for the development of tissue production and generation of carcinomas in different organs. Most probable expression in upper region of body and having effects in lungs, kidney displayed in Fig. 2. The presence of blue color indicates the high level regions among the bodies and gray color indicates the less expression and cutoff region in the body. The area of the mRNA directly upstream of the initiation codon is known as the 5′UTR. This area is involved in transcription control. Each mRNA transcript's comparable untranslated sections and corresponding positions were found. Tandem repeat elimination is a crucial step in the discovery of siRNA target locations.^35^

Typically, a tandem repeat consists of two or more neighboring copies of the same nucleotide sequence. The target site will not bind to siRNA if it ends with a length of four or five repetitions. Repeats are thus often eliminated throughout the siRNA design process. The 16S rRNA gene's mRNA straps are all unique and include no repeat, which calls for more investigation. Tissue-specific expression patterns highlight organs at higher risk of infection-related damage, aiding in the development of targeted diagnostic and therapeutic interventions for respiratory and systemic diseases.

### Design and probability assessment of hairpin siRNAs targeting 16S rRNA

Every siRNA is a well-known and distinct one, although all mRNAs lack a homology search in the NCBI. Then, we investigate the structural feature, CFTR, and 16S rRNA in the homology search; nevertheless, none of the genes with comparable sequences that are identified as anticipated target sites. Avoiding homologous target sites is essential because there is a possibility that the siRNA or shRNA that is being created will bind to the target site of the normal gene and inhibit its production if a similar sequence is found in any gene that serves as a target site. Different criteria for siRNA design were used by experts who initially reported using siRNA expression vectors to induce RNA interference. Two inverted repetitions separated by a brief buffer sequence constituted a significant portion of the designs, which concluded with a string of Ts acting as the translation end site.^36^ The length of the nucleotide arrangement being used as siRNA varies to varying degrees. A few studies have employed the 19-nucleotide sequence as the siRNA.^37^ On the other hand, some research teams have employed siRNA that ranges in length from 21 to 25–29 nucleotides.^38^ It is discovered that all siRNAs of these various lengths may activate gene silencing.^39^ 19 nucleotides in length were used to design the siRNA used in this study; 15 distinct siRNA were created for each target site. For further details, see the top-ranked siRNA reselection. In Table 2, the siRNA are displayed. Table 2's stated probability values for effective siRNA are excellent. The server's overall positive predictive value is 0.954, which indicates that 78.6% of the siRNAs it chooses will effectively silence targets. Testing against a database of siRNA studies carried out under various experimental settings yields the positive predictive value. High predictive silencing probabilities support the translational feasibility of these siRNAs as therapeutic candidates, increasing confidence in their clinical efficacy against pathogenic gene expression.

**Table 2:** List of all siRNAs of predicted fifteen mRNAs. Having oligo position, Energy interoligo and intraoligo energies and probability efficient score towards the target silencing.

Pos.	Oligo (5'->3')	Overall	Duplex	Tm-Dup	Break-targ.	Intraoligo	Interoligo	End_diff	prefilter_score
		kcal/moL	kcal/moL	degC	kcal/moL	kcal/moL	kcal/moL	kcal/moL	
1	UAAAUAACCUCAAAUAGAC	−17.7	−24.8	72.2	−6.2	−0.1	−9.6	1.36	7
2	AUAAAUAACCUCAAAUAGA	−16.1	−23.2	69.7	−6.2	0	−9.8	0.8	8
1	UUAAUAACUUCGAAUAGCC	−22.1	−26.3	74.7	−2.4	−0.1	−11.7	2.33	8
2	AUUAAUAACUUCGAAUAGC	−19.9	−24.1	70.7	−2.4	−0.1	−11.8	2.32	9
1	UAAACACCUUCAAAUAGAC	−22.3	−26.7	75.4	−3.4	−0.3	−9.7	1.36	7
2	AUAAACACCUUCAAAUAGA	−20.6	−25.1	73.1	−3.5	−0.2	−9.8	0.8	9
1	UUAAUAACUUCGAAUAGCC	−21.6	−26.3	74.7	−2.9	−0.1	−11.7	2.33	8
2	AUUAAUAACUUCGAAUAGC	−19.4	−24.1	70.7	−2.9	−0.1	−11.8	2.32	9
1	UUAACAACUUCUAAUAGCC	−20.1	−26.8	76.3	−5.8	−0.1	−9.8	2.33	7
2	AUUAACAACUUCUAAUAGC	−17.6	−24.6	72.2	−5.8	−0.2	−10.3	2.32	9
1	UUAAUAACUUCGAAUAGCC	−16.9	−26.3	74.7	−7.6	−0.1	−11.7	2.33	8
2	AUUAAUAACUUCGAAUAGC	−14.6	−24.1	70.7	−7.7	−0.1	−11.8	2.32	9
1	UUAAUAACUUCAAAUAGCC	−19.2	−24.5	72.2	−4.4	−0.1	−9.8	2.33	7
2	AUUAAUAACUUCAAAUAGC	−16.8	−22.3	67.9	−4.4	−0.1	−10.1	2.32	9
1	UUAAUACCUUCAAAUAGAC	−19.1	−24.8	72.2	−4.5	−0.3	−10.2	1.76	7
2	AUUAAUACCUUCAAAUAGA	−17.5	−23.2	69.7	−4.5	−0.2	−10.3	0.8	3
1	CUUAUAACUCCAAGUAGAC	−20.4	−28.8	78.1	−6.8	−0.5	−11.1	0.16	6
2	ACUUAUAACUCCAAGUAGA	−18.9	−27.8	78	−7	−0.9	−11.9	0.11	9
1	AUAAUACCUCCAAAUAGCC	−23	−29.5	79.6	−5.6	0	−9.5	2.16	8
2	AAUAAUACCUCCAAAUAGC	−20.5	−27.1	76	−5.7	0	−9.6	2.49	10
1	UAAAUAACUUCUGAUAGAC	−17.5	−25.1	72.1	−6.2	−0.3	−10.7	1.36	7
2	AUAAAUAACUUCUGAUAGA	−16.1	−23.5	69.7	−6.2	−0.2	−10.3	0.8	9
1	UUAAUACCUUCAAAUAGAC	−20	−24.8	72.2	−3.6	−0.3	−10.2	1.76	7
2	AUUAAUACCUUCAAAUAGA	−18.4	−23.2	69.7	−3.6	−0.2	−10.3	0.8	9
1	UUUAAAACUCCAAAUAGAC	−17.6	−24.2	71.5	−5.3	−0.1	−10.6	1.76	6
2	UUUUAAAACUCCAAAUAGA	−14.9	−22.4	69	−6.2	−0.1	−10.6	0.97	8
1	UUUAUAACUACAAACAGAC	−20.3	−24.5	72.3	−3	0	−10.3	1.76	6
5	ACUCUUUAUAACUACAAAC	−20.6	−24.5	72.4	−3.1	0	−9.2	0.9	6
25	AUUAUCAGUGGGCAGGCCA	−22	−36.5	89.8	−9.7	−2.4	−17.8	0.56	6
26	AAUUAUCAGUGGGCAGGCC	−22.8	−35.8	88.2	−9.6	−0.8	−14.9	2.33	8
**Position on target**		**Probability of being efficient siRNA**				**siRNA Sequence (**5'**->**3'**)**			
448		0.954373				UUUUAAUUCAACAUCGAGG			
446		0.942616				UUAAUUCAACAUCGAGGUC			
1		0.964323				UUAAUAACUUCGAAUAGCC			
446		0.953265				UUUUAAUUCAACAUCGAGG			
221		0.966146				UUUCUUUUACUCAUAAAGC			
1		0.955361				UAAACACCUUCAAAUAGAC			
1		0.962859				UUAAUAACUUCGAAUAGCC			
726		0.958892				UACUAACUCCACUAAUAAC			
340		0.967586				UUAUCGUCUACUCAGUCAC			
1		0.96452				UUAACAACUUCUAAUAGCC			
799		0.967749				UAAAAUCUUACGUUCAAGC			
818		0.961685				UACGAAUCUGAAUAUAACC			
1		0.963182				UUAAUAACUUCAAAUAGCC			
220		0.96186				UUAACCUCAUGUAUAAAGC			
152		0.968876				UAAAUUCUAAUAUAAUAGC			
560		0.958369				UUAUCAAGAGAUAGAAACC			
1052		0.966982				UAAACACCUAGACUCAAGC			
723		0.966018				UACAAACUCUACUAACAAC			
753		0.973651				UACACAUACAACUAAAACC			
1055		0.966403				UACACACCUAGUCUCAAGC			
1068		0.962603				UACUAUUACUCUAUAAACC			
735		0.962365				UAAAUAAUUCACGAACUCC			
152		0.975916				UAAAUUCUACUAUAACAGC			
1076		0.967948				UACUAUCACACUAUACACC			
722		0.969171				UUAUCACAAAAUAAUUAGC			
651		0.963422				UCUAACACAUAUAAUCUGC			
716		0.972748				UUAUCACAAAACAAUUAGC			
1		0.966383				UUUAUAACUACAAACAGAC			
1062		0.965227				UACACACUUAUACUUAAGC			
962		0.961492				UAAAUAAACUAAAUACAGG			

Since all of the likelihood values in this instance are higher than 0.8, it is certain that the anticipated siRNAs will be more successful in silencing the mutant 16S rRNA gene. Several research teams have demonstrated successful hairpin siRNA gene silencing using a loop of three to twenty-three nucleotides.^40^ According to our findings, all siRNAs have a probability larger than 0.8, indicating that they are a superior option for silencing a gene's impact. Typically, a hairpin siRNA is created with the target's sense strand first, then its antisense strand in a certain order, separated by a brief spacer. For every target site sequence, a hairpin siRNA was created. If the planned hairpin construct does not start with a purine at siRNA exert 3, an additional 'G' is inserted to the construct's beginning. It is preferred by RNA Poly Ⅲ to use a purine to start transcription. Typically, siRNA transports the siRNA duplex directly to the cytosol, where it may bind DNA. It is composed of two complementary 19–22 bp RNA sequences joined by a brief loop of 4–11 nucleotides, akin to the hairpin present in actual miRNA.

### Structural characterization of CFTR NBD1 and infection-related proteins

**Fig. 3:**
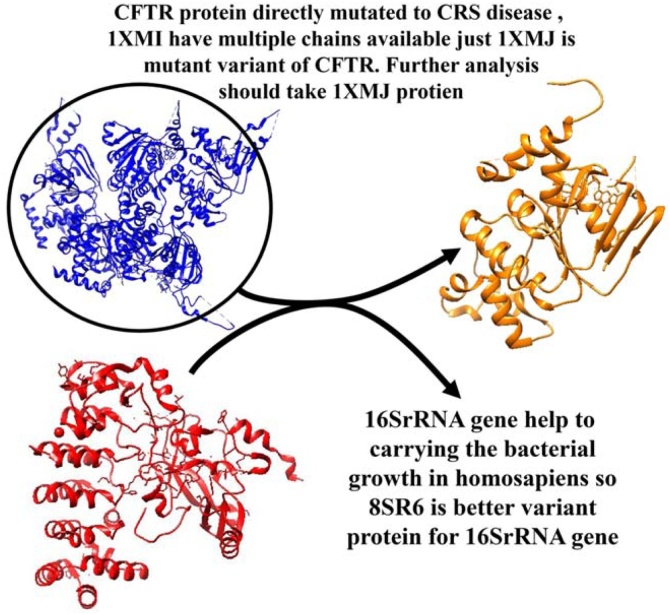
Illustrate the features and appearance of protein IXMJ having single chain available that retrieved from the IXMI multiple mutant protein of CFTR component hiving directly proportion to CRS illness, 16SrRNA gene translate into 8SR6 protein component.

The protein 1XMI, which have seven chains and a structure between 2 and 2.3 X-Ray crystallography score, is encoded to the CFTR protein complex that directly associate in CRS illness. Crystal structure of NBDI domain associated single chain A 1XMJ (Crystal structure of human F508A NBD1 domain with ATP). Both 1XMJ and 1XMI discovered in same year in 2005 for the component factor for CFTR in homosapiens.^41^ 1XMJ contain normal one chain taking for the further analysis, having structural conform changes Alpha helix, Beta sheets, coil and loop region available in it. The protein 8SR6 (Crystal structure of legAS4 from *Legionella pneumophila* subsp. pneumophila with histone H3 (3–17) peptide),^42^ which aids in translation, is directly responsive to 16SrRNA gene coding. The 8SR6 protein mutation directly affects the bacterial infection and help in spreading to all the body. The protein 8SR6 directly connect to Eukaryotic huntingtin interacting protein B in homosapiens which predicted in species *Legionella pneumophila* subsp. pneumophila. The structural features of the 1XMJ and 8SR6 proteins are explained in Fig. 3. Understanding the structural properties of CFTR and bacterial translation-associated proteins provides molecular insight into infection susceptibility and chronic respiratory disorders, supporting structure-guided therapeutic development.

### Functional annotation and association network analysis of CFTR and infection-related proteins

Our understanding of the molecular causes of human disease is nevertheless hampered by the persistent incompleteness of the data, despite extraordinary experimental efforts to map out the human interactome. An encouraging substitute is provided by computational techniques, which aid in the discovery of physiologically meaningful but as-yet-unmapped Protein-Protein Interactions (PPIs). Although biological or network-based similarity is the basis for link prediction approaches to connect proteins, comparable proteins do not always interact and interacting proteins are not always similar. Here, we present structural and evolutionary evidence supporting the idea that proteins interact when one of them is similar to the partner of the other rather than when they are similar to one other. Ozger identified the SARS-COVID-2 network route, its interactions with other proteins, and its effects on other proteins.^43^, ^44^, ^45^ Our study looks into the various biological functions and pathways that the 16S rRNA (8SR6) protein participates in, such as mRNA pseudouridine synthesis, mitochondrial ribosome assembly, enzyme-directed rRNA pseudouridine synthesis, assembly of the large ribosomal subunit in the mitochondria, and positive regulation of mitochondrial translation. It culminated in rRNA binding, rRNA methyltransferase activity, pseudouridine synthase activity, and rRNA (guanosine-2-O-)-methyltransferase activity on a molecular level. It coordinated several pathways inside the cell, including the mitochondrial nucleoid, ribosome, matrix, granule of ribonucleoprotein, and mitochondrion.

**Fig. 4:**
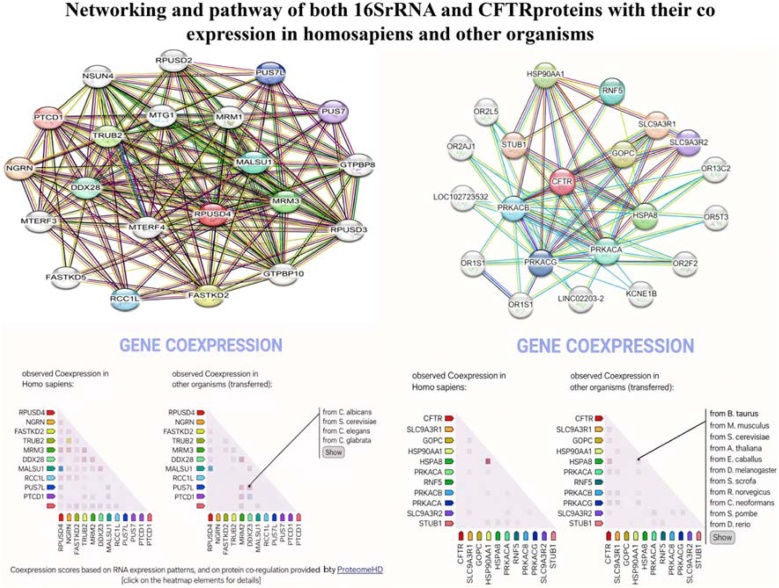
Explain the networking and pathway module of both 16SrRNA and CFTR proteins. Both proteins participate in various pathways and their co-expression lies in homosapiens and other organisms with great interest score.

The biological functions and pathways that the CFTR (1XMJ) protein is involved in vary. These include chaperone-mediated autophagy, protein kinase A signaling, high-density lipoprotein particle assembly, negative regulation of the smoothened signaling pathway involved in dorsal/ventral neural tube patterning, and regulation of anion channel activity.^46^ AMP-activated protein kinase activity, Type 2 metabotropic glutamate receptor binding, Type 3 metabotropic glutamate receptor binding, cAMP-dependent protein kinase activity, and TPR domain binding were the molecular outcomes. It synchronized many pathways with cAMP-dependent protein kinase complex, ciliary base, plasma membrane, and membrane in a biological manner. The co expression and network module of the CFTR protein (1XMJ) and 16S rRNA (8SR6) are described in Fig. 4. Mapping interaction networks between bacterial and host proteins enhances understanding of disease mechanisms and supports the identification of novel intervention points for complex infectious and genetic disorders.

### Implications for disease mechanisms and therapeutic targeting

**Fig. 5:**
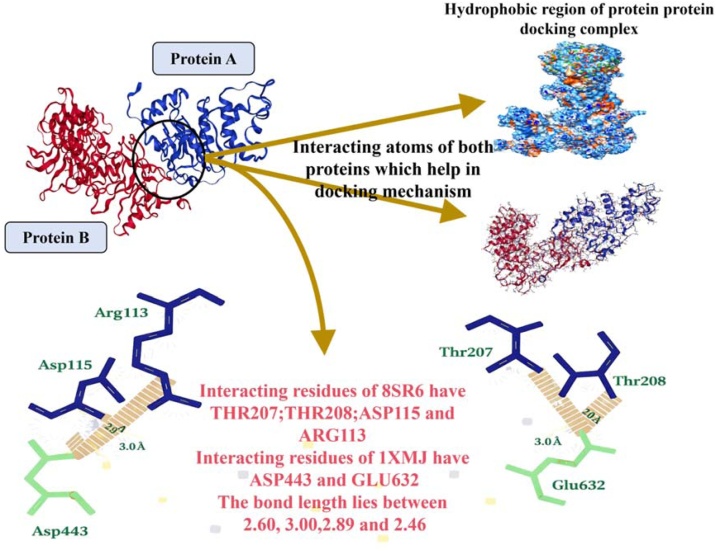
Describe the hydrophobic region, docking phenomenon of both proteins. Both proteins have interacted each other with high binding energy and distances.

The scientific community became interested in studying the properties of proteins because of their biological relevance. The research provided insight into how proteins interact and serve many purposes in a live organism.^47^ Protein-Protein Interactions (PPIs) have a variety of vital functions in cells, including those of protein-protein inhibitors, antibody-antigen complexes, and super complexes. It is amazing how structural analysis techniques, such as cryo-EM, have advanced recently for determining protein complex structures.^48^ When compared to C6 rat glioblastoma cells, naringin exhibits a greater cytotoxic potency against U-87MG human glioblastoma cells, suggesting that it may be used as a therapeutic treatment for glioblastoma.[53] Our research concluded that accession key 1XMJ protein for CFTR and 8SR6 protein for 16SrRNA display docking interaction. The 8SR6 protein treat as a receptor having active residue, which bind to other protein with high affinity; THR207; THR208; ASP115 and ARG113. Similarly, 1XMJ protein treat as ligand in this complex shown their interaction residues; ASP443 and GLU632. Both proteins (ligand + Receptor Complex) interact each other with lowest energy -844.0 and -895.9 and bond lies at center of residues having distance noticed. This protein-protein interaction complex cluster the residues of both proteins having distance 2.60, 3.00, 2.89 and 2.^42^ Fig. 5 illustrate the docking interaction, hydrophobic and distance between the bonds of interacting residues. This integrative approach bridges molecular biology and clinical application, offering a rational pathway toward precision RNA therapeutics for infection-related and genetic diseases.

## Conclusion

This study demonstrates the successful computational design of siRNA targeting 16S rRNA variants in Chronic Rhinosinusitis (CRS), with high silencing probability (> 0.8) and no off-target homology. Protein-protein docking revealed strong inhibitory interactions between mutant CFTR (1XMJ) and 16S rRNA-associated protein 8SR6 (ΔG = -895.9 kcal/moL), highlighting a novel therapeutic pathway.

## ORCID ID

Jiaoyu He: 0000-0003-4585-1354

Jiaxin Li: 0009-0000-5365-580X

Feng Liu: 0000-0003-2866-2340

## Author' contributions

Bo Wei was sresponsible for the conceptualization, methodology, writing-original draft; Jiaoyu He was responsible for the writing-review & editing; Jiaxin Li was responsible for the data curation, formal analysis, investigation; Feng Liu were responsible for the conceptualization.

## Data availability statement

The authors declare that all data are available in repository.

## Declaration of competing interest

The authors declare no conflicts of interest.
